# Somatic comorbidity among migrants with posttraumatic stress disorder and depression – a prospective cohort study

**DOI:** 10.1186/s12888-016-1149-2

**Published:** 2016-12-13

**Authors:** Mette Lolk, Stine Byberg, Jessica Carlsson, Marie Norredam

**Affiliations:** 1 Danish Research Centre for Migration, Ethnicity and Health, Section of Health Services Research, Department of Public Health, University of Copenhagen, Øster Farimagsgade 5A, Copenhagen, Denmark; 2 Competence Centre for Transcultural Psychiatry, Mental Health Centre Ballerup, Mental Health Services of the Capital Region of Denmark, Maglevaenget 2, 2750 Ballerup, Denmark; 3 Department of Immigrant Medicine, Section of Infectious Diseases, Hvidovre Hospital, Kettegård Allé 30, Copenhagen, Denmark

**Keywords:** Post-traumatic stress disorder, Depression, Comorbidity, Refugee, Migrant

## Abstract

**Background:**

In a cohort of migrants in Denmark, we compared somatic disease incidence among migrants diagnosed with posttraumatic stress disorder (PTSD) and depression with migrants without a diagnosed psychiatric disorder.

**Methods:**

The study builds on a unique cohort of migrants who obtained residence permit in Denmark from 1993 to 2010 (*N* = 92,104). The association with somatic disease was explored via register linkage. We used Poisson regression to model incidence rate ratios (IRR) adjusted for age, sex, income and region of origin. The Danish Data Protection Agency granted authorisation for the implementation of the project (No 2012-41-0065).

**Results:**

Our results showed that migrants diagnosed with PTSD and depression had significantly higher rates of somatic diseases compared with migrants without diagnosed psychiatric disorders – especially, infectious disease (IRR, 1.89; 95% CI, 1.45–2.48; *p* < 0.01), neurological disease (IRR, 2.35; 95% CI, 1.91–2.91; *p* < 0.01) and pulmonary disease (IRR, 1.69; 95% CI, 1.37–2.00; *p* < 0.01). We further saw differences in the IRRs according to region of origin.

**Conclusion:**

Migrants with PTSD and depression had a significantly higher rates of somatic comorbidity compared with migrants without a diagnosed psychiatric disorder. The rates were especially high for infectious, neurological and pulmonary diseases. Our results further suggest difference in the rates of somatic comorbidity according to region of. Preventive and treatment services should pay special attention to improve the overall health of migrants with PTSD and depression.

## Background

In 2015, 244 million people worldwide, equalling 3.3% of the world’s population, were considered international migrants. Globally, in 2015, there were an estimated 19.5 million refugees and 1.8 million asylum seekers [1]. In 2015, 8.9% of the Danish population was classified as international migrants of whom 58% originated from non-Western countries. The majority of non-Western international migrants to Denmark were refugees or family reunified individuals [2]. In the last four years, the number of asylum seekers in Denmark has increased more than fivefold and the number of family reunified migrants in Denmark has increased fourfold [3].

Refugees often experience traumatic events associated with war, persecution, torture, sexual violence and the challenges of resettling in exile [4, 5]. It is well-established that trauma-affected refugees are at risk of developing posttraumatic stress disorder (PTSD) and depression [6–8]. For a large number of these refugees the psychiatric disorders often become chronic despite intensive therapy [9, 10]. In general, patients suffering from PTSD self-report a high rate of general medical complaints [11–14] and numerous studies have found an association between PTSD and several somatic disorders; in particularly cardiovascular diseases and diabetes mellitus [15–17]. The majority of these studies however, have focused on war veterans and the relationship between somatic disease and PTSD among migrants has received less attention [18–21]. Furthermore, the causal links between PTSD and somatic comorbidities are still unknown [12, 14, 19]. With increasing migration as described above, migrant health has become an increasing concern [22]. Understanding the magnitude and causal mechanisms of the somatic disease burden among migrants with PTSD is crucial to ensuring better health outcome for the patients and also to implement appropriate preventive measures at an early stage. Migrants may differ in disease burden compared with local born, due to both genetics, cultural/life style factors and life course factors [23] and therefore the most relevant comparison group for assessing excessive somatic comorbidity in migrants with diagnosed PTSD, should be migrants originating from the same areas as the trauma-affected migrants.

Research on trauma-affected refugees as well as our clinical experience with the target group show that a very large proportion of refugees suffering from PTSD also suffer from comorbid depression [8]. The number of trauma-affected refugees with PTSD and comorbid depression was as high as 94% in a clinical sample at Competence Centre for Transcultural Psychiatry (CTP) [14]. Like PTSD, depression is also known to be associated with a number of somatic diseases [21, 24, 25]. Taking into consideration the substantial degree of comorbidity between PTSD and depression, we decided to look at comorbid PTSD and depression in the present study as we could not disentangle the two.

Consequently, in an exploratory study, we investigated the association between somatic disease among migrants (refugees and family reunified) diagnosed with PTSD and depression and migrants without a diagnosed psychiatric disorder in a large national cohort of all refugees and family reunified migrants who obtained residence in Denmark during an 18-year period.

## Methods

### Population

**Fig. 1:**
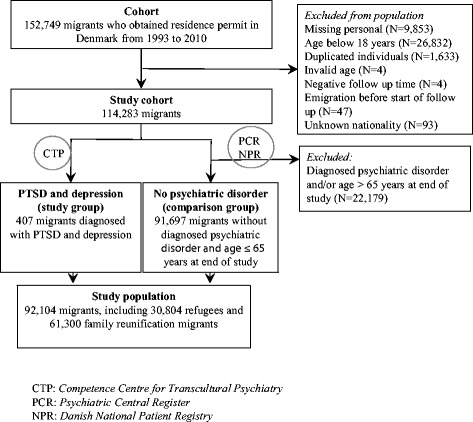
The study cohort

We conducted a historically prospective cohort study. In collaboration with the Danish Immigration Service we established a unique, large cohort comprising all migrants who obtained right of residence as refugees or through family reunification in Denmark between 1 January 1993 until 31 December 2010. By register-linkage, we were able to prospectively follow the migrants in the various Danish registers from the date of being granted residence permit. We included refugees and individuals who came to Denmark through family reunification because many are family reunified to refugees (others are reunified to immigrants or Nordic citizens). Furthermore, this decision was built on that family reunified to refugees are an understudied group and often have had a similar life course including traumatic events, to refugees and thus, might also be at higher risk of PTSD and depression. In total, 152,749 migrants were identified. Migrants were excluded for the following reasons: i) missing personal identification number, ii) age at residence below 18 years, iii) duplicated individuals, iv) invalid age, v) negative follow-up time (diagnosis made before date of residence permit), vi) emigration before start of follow-up or vii) unknown nationality. Consequently, the final study cohort comprised 114,284 migrants (Fig. 1).

Data derived from the Danish Immigration Service contained eight variables: nationality, sex, birth year, foreign number, personal identification number (PIN), date of entry, date of residence permit and legal background of residence permit. Every citizen in Denmark has a PIN that can be used to identify persons through public registers at the individual level [26]. For newly arrived immigrants, the PIN is assigned together with the residence permit. The cohort was linked to several national registers using the PIN.

The Danish Data Protection Agency granted authorization for the implementation of the project (No 2012-41-0065).

#### Defining migrants with PTSD and depression (study group)

To ensure the most valid diagnosis of PTSD and depression among individuals in the cohort we linked the cohort to the migrant patient population at the Competence Centre for Transcultural Psychiatry (CTP). At the time of this study approximately 200 patients were in treatment at CTP yearly [27]. We included all patients at CTP diagnosed with PTSD and depression in the study. CTP is the largest clinic in Denmark specializing in psychiatric treatment of migrants with trauma related disorders. Patients can be referred to CTP by all general practitioners, psychiatric practitioners or medical doctors at a psychiatric or somatic hospital. First time diagnoses of PTSD and depression were determined by a trained medical doctor after a three-hour preliminary examination [28], where ICD-10 research criteria for each of the diagnoses were entered in a diagnostic algorithm [29]. Psychotic and bipolar disorders were excluded using parts of the Schedules for Clinical Assessment in Neuropsychiatry (SCAN), version 2 [30]. All doctors performing these interviews were certified SCAN raters. Through a match of the CTP database and our cohort, a total of 407 persons from our initial study cohort were identified as having been diagnosed with both PTSD and depression at CTP and, thus, defined as the study group: *PTSD and depression* (Fig. 1).

#### Defining migrants with no psychiatric disorder (comparison group)

Migrants in the study cohort not diagnosed with a psychiatric disorder were defined as the comparison group: *No psychiatric disorder*. Migrants with any psychiatric disorder registered in the Psychiatric Central Register (PCR) (ICD-10 code F00-F99) not limited to PTSD and depression were excluded for two main reasons: PTSD can fluctuate over time and mimic other anxiety disorders or/and other psychiatric disorders [6]. Thus, to try and avoid including any persons with PTSD and depression in the comparison group, we excluded all migrants with a psychiatric diagnosis. Second, there are several other psychiatric disorders that are associated with somatic disease, which would underestimate the association between PSTD, depression and somatic disease [31]. The Psychiatric Central Register (PCR) contains data on all psychiatric admissions from 1 January 1969, including outpatient contacts and psychiatric emergency room visits since 1 January 1995 [32]. Since we found a relatively low age in the study group *PTSD and depression* at the end of study (age < 63 years), we decided to exclude persons older than 65 years from the comparison group *No psychiatric disorder*. Chronic somatic diseases are correlated with increasing age [33] and, therefore, could bias the disease estimates if the age distribution in the groups were markedly different. In total, 91,697 migrants were included in the comparison group: *No psychiatric disorder* (Fig. 1).

### Data collection

We defined somatic comorbidity as all ICD-10 diagnoses made at a Danish hospital except for the psychiatric diagnoses, ICD-10 chapter V (F00-F99) and diagnoses involving pregnancy, conditions originating in the perinatal period, congenital malformations, symptoms and injuries, ICD-10 chapter XV to XXI (O00-Z99).

We obtained information on somatic comorbidity as well as psychiatric diagnoses by linking migrants to the Danish National Patient Registry (NPR), the Danish National Diabetes Register (NDR) and the Danish Psychiatric Central Register (PCR) via their personal identification number (PIN). The NPR holds information on all hospital-registered diagnoses and hospital contacts on all inpatients in Denmark since 1 January 1977, including outpatient and emergency visits since 1 January 1995. Every single patient contact has at least one diagnosis code, and the reporting is obligatory [34]. The NDR includes both hospital diagnosed diabetes mellitus (DM) as well as DM diagnosed in primary care provided by general practitioners. The register is more than 90% complete and covers the entire Danish population [35]. Furthermore, we obtained data on date of emigration, emigration destination and death from the Danish Civil Registration System (CRS) [36] and annual personal income from Statistics Denmark (DST) [37].

### Variable definition

Somatic disease during the study period was the main outcome. Data on hospital diagnoses were obtained from NPR. Primary, secondary and supplementary hospitals diagnoses were included to obtain as much information on diagnoses as possible. The outcome, somatic disease, was divided into fifteen different diagnostic categories according to ICD-10 general classification (see table 2). Diagnostic category fourteen *Any somatic ICD-10 diagnosis (A00-N99),* was created by combining all previous categories from one to thirteen. Diagnostic category number fifteen *Diabetes mellitus (from NDR)*, was based on supplementary details on diabetes diagnoses from the NDR. Only diagnoses registered after date of residence was included in the present study.

Migrant status was defined on the basis of background of residence permit and divided into four groups; 1) quota refugees (obtained asylum prior to entering the new country following an agreement with the Office of the United Nations High Commissioner for Refugees (UNHCR)), 2) spontaneous asylum seekers, 3) family reunification with refugee, and 4) family reunification to non-refugee, including family reunification with immigrant or family reunification with Danish or Nordic citizen. Persons in the study cohort were divided into five large geographic groups based on country of birth; 1) Eastern, Central and Southern Asia, 2) Europe, 3) Sub-Saharan countries, 4) Western Asia and North Africa, and 5) Other incl. America and Oceania, according to the United Nations Statistics Division [38].

### Statistical analyses

We tested for sex differences in socio-economic characteristics using chi-square test and student’s *t*-test. Separate analyses were conducted for each of the ICD-10 categories; all persons in the study cohort, both migrants with and without PTSD and depression, were followed from date of obtaining residence permit until one of the following events: 1) date of first diagnosis within the ICD-10 category being analysed, 2) date of first emigration, 3) death, or 4) end of study 31 December 2011, whichever came first. Persons obtaining residence permit before 1 January 1994, where followed from this date. We calculated separate follow-up time for each ICD-10 category as migrants could have more than one disease and therefore could be censored at different times depending on the disease outcome.

We adjusted for income because low income is considered a risk factor for excess somatic and psychiatric disease. The mean annual personal income was calculated from income one year after obtaining residence until one year before end of study and allocated into a categorical income variable. Mean annual income was therefore calculated separately for each of the fifteen diagnostic categories as censoring of follow-up varied between diagnostic category groups. Mean annual incomes were divided into four categories; 1) ≤ 9999 Euro, 2) 10,000–19,999 Euro, 3) 20,000–29,999 Euro, and 4) ≥ 30,000 Euro based on analysis of the income distribution.

Incidence rates (IR) for individual hospital diagnosis for primary, secondary and supplementary diagnoses were estimated as cases per 100,000 person years at risk with a 95% confidence interval. The association between PTSD, depression and somatic disease was assessed by Poisson regression analysis. Confounder control was done using a staged approach. The first model comprised the unadjusted association. The second model was adjusted for sex and age. The third model was further adjusted for annual income. The fourth model added region of origin. We decided also to stratify the adjusted model by region of origin and by basis of residence for seven out of the fifteen categories, as the seven categories had a sufficient number of cases to conduct further analysis. A *p*-value of < 0.05 was considered statistically significant. SAS version 9.2 (SAS Institute, Cary, NC, USA) was used for all analyses on the research platform provided by Statistics Denmark in accordance with the laws passed by the Danish Data Protection Agency [37]. The Danish Data Protection Agency approved the study. Further ethical approval for registry-based research is not required in Denmark [39].

## Results

### Characteristics of the study cohort

The characteristics of the study cohort are presented in Table 1. The median age at residence was 34 years for men and 32 years for women among migrants with PTSD and depression. For migrants without psychiatric disorder the median age at residence were 30 and 29 years for men and women, respectively. Migrants with PTSD and depression were followed for 12 years on average whereas migrants without were followed for 11 years on average. For both groups, women were significantly more likely to be family reunified compared to men. The highest percentages of migrants with no psychiatric disorder were in the group of migrants who were family reunified to non-refugees. Migrants with PTSD and depression mainly originated from Afghanistan (12%), Iran (7%), Former Yugoslavia (17%), Iraq (44%) or were Stateless Palestinians (5%). In comparison, 38% of the migrants without a diagnosed psychiatric disorder originated from one of these five places.

**Table 1:** Characteristics of the study cohort

	PTSD and depression (study group)				No psychiatric disorder (comparison group)			
	*N* = 407				*N* = 91,697			
Percentage (%)	All	Men	Women	*P*-value^a^	All	Men	Women	*P*-value^a^
Gender		48.6	51.4			40.7	59.3	
Region of origin				>0.05				<0.01
Eastern, Central and South. Asia	20.4	21.7	19.1		27.1	19.2	32.5	
Afghanistan	12.3	9.6	14.8		9.5	5.4	12.3	
Iran	7.1	10.1	4.3		2.8	2.9	2.7	
Europe	18.9	18.2	19.6		34.7	36.5	33.5	
Former Yugoslavia	16.5	18.2	15.3		15.0	19.0	12.3	
Sub-Saharan countries	5.2	4.5	5.7		13.1	13.8	12.0	
Western Asia and North Africa	55.5	55.6	55.6		17.0	21.2	14.1	
Iraq	43.5	42.4.	44.5		8.9	11.6	7.1	
Stateless Palestine	5.4	5.1	5.7		2.0	2.1	1.9	
Other incl. America and Oceania	0.0	0.0	0.0		8.1	8.4	7.9	
Basis for residence				<0.01				<0.01
Refugee	74.4	89.4	60.3		33.3	49.0	22.4	
Asylum seeker	67.3	79.8	55.5		29.2	42.9	19.7	
Quota refugee	7.1	9.6	4.8		4.1	6.1	2.7	
Family reunified	25.6	10.6	39.7		66.7	51.0	77.6	
To refugee	19.2	5.1	32.5		10.9	5.5	14.6	
To non-refugee	6.4	5.6	7.2		55.8	45.4	63.0	
Events during follow up				>0.05				<0.01
Emigrated	5.2	6.6	3.8		16.6	18.0	15.6	
Death	0.2	0.5	0.0		1.1	1.6	0.8	
End of study	94.6	92.9	96.2		82.3	80.4	83.6	
Annual income				<0.01				<0.01
≤9.999 EUR	3.2	5.1	1.1		25.4	17.4	30.9	
10.000–19.999 EUR	30.5	50.8	8.3		27.6	30.7	25.4	
19.000–29.999 EUR	35.0	35.0	35.0		30.3	28.3	31.7	
≥30.000 EUR	31.3	9.1	55.6		16.6	23.5	11.9	
Median (first - third quartile)		Men	Women	*P*-value^a^		Men	Women	*P*-value^a^
Age at residence (years)		34	32	>0.05		30	29	>0.05
		(28–39)	(27–39)			(25–36)	(25–35)	
Age end of study (years)		45	45	>0.05		41	39	>0.05
		(8.86)^b^	(8.7)^b^			(35–47)	(33–46)	
Follow up time (years)		12	12	>0.05		11	10	>0.05
		(9–16)	(6–16)			(6–16)	(5–14)	

^a^Chi-square test

^b^Mean (SD)

### Incidence rate of somatic comorbidities

**Fig. 2:**
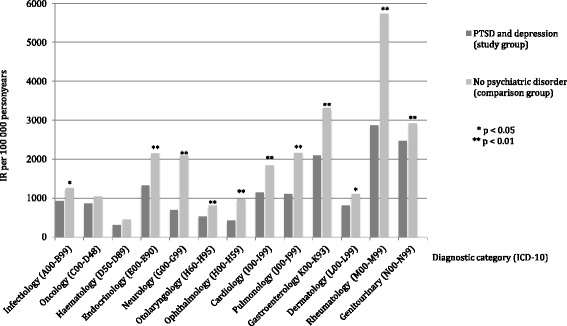
Incidence rate (IR) of hospital diagnosed somatic disease (ICD-10) among migrants with PTSD and depression (*N* = 407) and migrants without diagnosed psychiatric disorder (*N* = 91,697)

Figure 2 shows the incidence rates (IR) for somatic hospital diagnoses for the selected diagnostic categories. The IR was significantly higher for all diagnostic categories except for oncology and haematology among migrants with PTSD and depression compared to migrants without.

### Adjusted incidence rate ratio for somatic comorbidities

Table 2 shows incidence rate ratio (IRR) of somatic hospital diagnoses among migrants with PTSD and depression compared to migrants without psychiatric disorder. In general, the IRR’s were attenuated, but still significant, when adjusted for age, sex, annual income and region of origin. In the final model, the IRR of ten out of fifteen diagnostic categories were significantly higher among migrants with PTSD and depression compared with migrants without psychiatric disorder. The IRR was especially high for infectious diseases (IRR, 1.89; 95% CI, 1.45–2,48; *p* < 0.01), neurological diseases (IRR, 2.35; 95% CI, 1.91–2.91; *p* < 0.01) and pulmonary diseases (IRR, 1.69; 95% CI, 1.37–2.00; *p* < 0.01).

**Table 2:** Incidence rate ratio (IRR) for hospital diagnosed somatic disease among migrants with PTSD and depression (*N* = 407) and migrants without diagnosed psychiatric disorder (*N* = 91,697)

	Proportion	IRR (95% CI)			
Diagnosis (ICD-10 code)	% (N)	Model 1	Model 2	Model 3	Model 4
No psychiatric disorder		1.00	1.00	1.00	1.00
PTSD and depression					
Infectiology (A00-B99)	13.8 (56)	1.36 (1.05–1.77)*	1.41 (1.10–1.84)**	1.61 (1.12–2.10)**	1.89 (1.45–2.48)**
Oncology (C00-D48)	11.8 (48)	1.21 (0.91–1.61)	1.12 (0.84–1.48)	1.24 (0.93–1.65)	1.30 (0.97–1.73)
Haematology (D50-D89)	5.2 (21)	1.49 (0.97–2.30)	1.51 (0.98–2.32)	1.63 (1.05–2.54)*	1.44 (0.92–2.24)
Endocrinology (E00-E90)	23.3 (95)	1.63 (1.33–1.99)**	1.62 (1.32–1.98)**	1.72 (1.40–2.11)**	1.43 (1.17–1.75)**
Neurology (G00-G99)	22.4 (91)	3.02 (2.45–3.71)**	2.79 (2.27–3.43)**	2.70 (2.18–3.33)**	2.35 (1.91–2.91)**
Otolaryngology (H00-H59)	10.8 (44)	1.53 (1.11–2.12)**	1.43 (1.04–1.98)*	1.44 (1.03–2.01)*	1.38 (0.99–1.92)
Ophthalmology (H60-H95)	9.1 (37)	2.32 (1.72–3.12)**	2.15 (1.60–2.90)**	1.94 (1.41–2.67)**	1.64 (1.19–2.27)**
Cardiology (I00-I99)	20.1 (82)	1.60 (1.29–1.99)**	1.41 (1.13–1.75)**	1.43 (1.15–1.77)**	1.36 (1.09–1.67)**
Pulmonology (J00-J99)	22.6 (92)	2.00 (1.60–2.42)**	1.97 (1.60–2.42)**	1.94 (1.57–2.39)**	1.69 (1.37–2.00)**
Gastroenterology (K00-K93)	32.7 (133)	1.58 (1.33–1.88)**	1.50 (1.26–1.78)**	1.46 (1.22–1.74)**	1.42 (1.19–1.69)**
Dermatology (L00-L99)	12.3 (50)	1.38 (1.04–1.82)*	1.40 (1.06–1.84)*	1.94 (1-41–2.67)**	1.27 (0.95–1.70)
Rheumatology (M00-M99)	53.3 (217)	2.00 (1.75–2.28)**	1.82 (1.59–2.08)*	1.77 (1.54–2.02)**	1.60 (1.40–1.84)**
Genitourinary (N00-N99)	29.5 (120)	1.18 (0.99–1.41)	1.26 (1.05–1.50)*	1.38 (1.45–1.66)**	1.29 (1.07–1.55)**
Any somatic ICD-10 diagnosis	86.2 (351)	1.29 (1.16–1.43)**	1.25 (1.12–1.39)**	1.29 (1.16–1.44)**	1.22 (1.09–1.36)**
Diabetes mellitus (from NDR)	7.6 (31)	1.54 (1.08–2.19)*	1.27 (0.89–1.81)	1.26 (0.88–1.82)	1.14 (0.79–1.64)

Model 1: Unadjusted

Model 2: Adjusted for sex and age

Model 3: Adjusted for model 2 + income

Model 4: Adjusted for model 3 + region of origin

* *p* < 0.05

** *p* < 0.01

### Region of origin

Table 3 shows IRR of somatic hospital diagnoses among migrants with PTSD and depression compared with migrants without psychiatric disorder, stratified by region of origin. Among migrants from Eastern, Central and Southern Asia, the incidence of endocrine, neurological, pulmonary and rheumatoid diagnoses were significantly higher among migrants with PTSD and depression compared with migrants without PTSD and depression. Among migrants of European descent, the incidence rates for neurological disease were significantly higher among migrants with PTSD and depression compared with migrants without PTSD and depression. This was also the case among migrants with PTSD and depression of Sub-Saharan origin compared with migrants without PTSD and depression, although the IRR was higher for migrants of Sub-Saharan descent (IRR, 2.85; 95% CI, 1.06–7.62; *p* < 0.05) relative to migrants of European descent (IRR, 1.76; 95% CI, 1.02–3.04, *p* < 0.05). All other disease categories were also higher among migrants with PTSD and depression of European or Sub-Saharan African descent, except for endocrine diseases, although not significant. Migrants with PTSD and depression from Western Asia and North Africa had a significantly higher IRR for six out of the seven diagnostic categories.

**Table 3:** Incidence rate ratio (IRR) for hospital diagnosed somatic disease among migrants with PTSD and depression (*N* = 407) and migrants without diagnosed psychiatric disorder (*N* = 91,697) adjusted for sex, age, income and stratified by region of origin

	IRR (95% CI)			
Diagnostic category (ICD-10)	Eastern, Central and Southern Asia	Europe	Sub-Saharan countries	Western Asia and North Africa
No psychiatric disorder	1.00	1.00	1.00	1.00
PTSD and depression				
Endocrinology (E00-E90)	2.55 (1.66–3.93)**	0.90 (0.47–1.73)	0.79 (0.30–2.11)	1.40 (1.08–1.81)*
Neurology (G00-G99)	2.98 (1.87–4.76)**	1.76 (1.02–3.04)*	2.85 (1.06–7.62)*	2.24 (1.70–2.95)**
Cardiology (I00-I99)	1.45 (0.87–2.41)	1.33 (0.86–2.07)	1.35 (0.43–4.19)	1.33 (0.98–1.79)
Pulmonology (J00-J99)	2.25 (1.43–3.55)**	1.22 (0.65–2.27)	1.95 (0.81–4.7)	1.63 (1.25–3.13)**
Gastroenterology (K00-K93)	1.32 (0.87–2.00)	1.17 (0.77–1.76)	1.35 (0.64–2.83)	1.53 (1.21–1.94)**
Rheumatology (M00-M99)	2.23 (1.67–2.98)**	1.23 (0.88–1.71)	1.45 (0.75–2.78)	1.58 (1.32–1.89)**
Genitourinary (N00-N99)	1.21 (0.76–1.93)	1.27 (0.83–1.95)	2.00 (1.00–4.01)	1.29 (1.01–1.65)*

IRR adjusted for age, sex and income

*p < 0.05

**p < 0.01

### Residence permit

Table 4 shows IRR of somatic hospital diagnosed disease among migrants with PTSD and depression compared with migrants without any psychiatric disorder, stratified by residence permit. Both refugees and family reunified migrants with PTSD and depression had significantly higher IRR for infectious, endocrine, neurological, pulmonary, gastrointestinal and rheumatic diseases compared with migrants without psychiatric disorder regardless residency. Family reunified migrants but not refugee migrants diagnosed with PTSD and depression had a significantly higher risk of cardiovascular and ophthalmological diseases compared with family reunified migrants with no psychiatric disorder.

**Table 4:** Incidence rate ratio (IRR) for hospital diagnosed somatic disease among refugees with PTSD and depression (*N* = 303), family reunified with PTSD and depression (*N* = 104) and migrants without diagnosed psychiatric disorder (*N* = 91,697) adjusted for sex, age, income and stratified by basis of residence permit

Diagnostic category (ICD-10)	IR (95% IC)	
	Refugees	Family reunified
No psychiatric disorder	1.00	1.00
PTSD and depression		
Infectiology (A00-B99)	1.55 (1.13–2.12)**	1.75 (1.06–2.91)*
Oncology (C00-D48)	1.22 (0.87–1.70)	1.18 (0.67–2.08)
Haematology (D50-D89)	1.39 (0.80–2.40)	1.90 (0.90–4.00)
Endocrinology (E00-E90)	1.35 (1.05–1.74)*	2.39 (1.70–3.37)**
Neurology (G00-G99)	2.35 (1.82–3.02)*	3.65 (2.48–5.37)**
Otolaryngology (H60-H95)	2.04 (1.44–2.88)**	1.21 (0.50–2.21)
Ophthalmology (H00-H59)	1.30 (0.87–1.95)	2.06 (1.14–3.3)*
Cardiology (I00-I99)	1.25 (0.97–1.60)	1.75 (1.11–2.75)*
Pulmonology (J00-J99)	1.75 (1.37–2.24)**	2.39 (1.61–3.54)**
Gastroenterology K00-K93)	1.32 (1.08–1.61)**	1.50 (1.02–2.21)**
Dermatology (L00-L99)	1.31 (0.94–1.84)	1.48 (0.84–2.61)
Rheumatology (M00-M99)	1.61 (1.38–1.88)**	1.99 (1.51–2.21)*
Genitourinary (N00-N99)	1.42 (1.14–1.77)**	1.25 (0.89–1.75)

IRR adjusted for age, sex and income

*p < 0.05

**p < 0.01

### Specific diagnosis

Exploring the most common specific diagnosis for five of the diagnostic ICD-10 categories for migrants with PTSD and depression shows the most common diagnoses in the category Cardiology (ICD-10 I00-I99) were; *ischemic heart disease (I20-25)* (7% of the migrants with PTSD and depression), *other forms of heart disease (I30-52)* (6%) including *paroxysmal infarction (I47)* and *atrial fibrillation and flutter (I48)*. The most common diagnoses in the categories *Infectious diseases (A00-B99)* were *intestinal infectious disease (A00-09*) (4%), *other viral diseases (B25-34)* and *viral hepatitis (B15-19)* (3%). Most common diagnoses in the category *Pulmonology (J00-99)* were *influenza and pneumonia (J09-18)* (7%). Most common diagnoses in the category *Endocrinology (E00-99)* were *diabetes mellitus (NDR)* (8%) and *obesity (E65–68)* (9%). Finally, the most common diagnoses in the category *Neurology (G00–99)* were *episodic and paroxysmal disorders (G40–47)* (16%) including *migraine (G40)*, *epilepsy (G43)*, *other headache syndromes (G44)*.

## Discussion

We found significantly higher rates of somatic comorbidity among migrants with PTSD and depression compared with migrants without a diagnosed psychiatric disorder. Adjusted rates were significantly higher in ten out of the fifteen diagnostic categories being especially high for infectious, neurological and pulmonary diseases. Our results further suggest difference in the rates of somatic comorbidity according to region of origin and according to the legal ground of obtaining residency.

Prior to our study, somatic comorbidity in migrants with PTSD and depression have only received scarce attention in the literature [18–21]. Albeit, diagnosis and treatment of somatic comorbidity may help improve management of mental disorders and vice versa. Further, patients with PTSD and depression suffering from somatic comorbidity will require regular treatment and check-up, such as diabetes may need tailored treatment programmes to ensure they are treated according to need. It is therefore important that somatic comorbidity is diagnosed and treated accordingly. Further, it is important to study characteristics of migrants with PTSD and depression and somatic comorbidity in order to identify patient groups most at risk and thereby, ensure adequate treatment response. There are, however, several arguments why studies are needed within this research area. First, migrants often have a long and complicated trauma history and exploratory studies show association between PTSD and chronic disease [16–18]. Second, there is growing evidence that patients with mental disease have worse outcomes for somatic morbidity and mortality which may be related to obstacles to the use of health care service [40, 41].

Based on evidence from previous studies, we hypothesized that migrants diagnosed with PTSD and depression would have higher rates of somatic comorbidity. Comorbidity between PSTD and somatic diseases in the general population has previously been described; thus prevalence of cardiovascular disease, arthritis, asthma, diabetes, epilepsies, liver- and kidney disease, thyroid disease, dermatological disease and metabolic syndrome has been shown to be higher in patients with PTSD [16–18], although the causal links remain unknown. A number of studies have examined potential biological mechanisms of the pathogenicity in PTSD. In patients suffering from PTSD the physiological response to stress alternate two major hormone system, the hypothalamic-pituitary-adrenal (HPA) axis and the sympathetic-adrenal-medullary (SAM) axis in the human body. Patients suffering from PTSD have an altered peripheral cortisol response during the day (less cortisol “spikes” upon awakening and a lowered cortisol level in the afternoon). These alterations seem to be associated with somatic diseases including cardiovascular disease and insulin resistance. Further, the immune response the patients suffering from PTSD are altered (increased inflammatory markers, lower total T lymphocyte count, lower natural killer cell activity). Both central and peripheral concentrations of catecholamines are elevated and SAM axis reactivated. Increased catecholamines and endothelia damage may lead to cardiovascular diseases. Notable, the somatic diseases associated to PTSD often has inflammatory or autoimmune underpinnings [42–44]. Some of these biological mechanisms, or a combination thereof, may explain the findings in our study; however, a confirmation of this theory requires more longitudinal studies of the neurobiological system.

Only few studies have explored the associations of PTSD and somatic comorbidity among migrants. A US study [20] found a higher prevalence of both DM and hypertension among traumatised refugees, especially those from Somalia, compared with the general US population. This is in accordance with our findings where migrants with PTSD and depression had higher rates of endocrine disease than migrants without PTSD and depression. Furthermore, we found that family reunified migrants but not refugees with PTSD and depression had a significantly higher rate of cardiovascular disease compared with migrants without psychiatric disorder. Likewise, a Dutch study [18] found a higher prevalence of type 2 DM among asylum seekers with PTSD compared with those without PTSD. The study however lacked an association between depression and type 2 DM. The influence of depression is uncertain and depression may serve as a surrogate marker for PTSD and not otherwise be independently associated with DM [45]. Another study demonstrated associations between PTSD and numerous different somatic diseases and found an overall relatively high rate of a variety of somatic complaints among refugees [19]. The study included both refugees suffering from PTSD, depression and other psychiatric disorders. However, the medical complaints and diseases were self-reported. Nevertheless, this study as well as this present study might support the hypothesis that an association between PTSD, depression and somatic disease includes a variety of different somatic diseases. The relationship between PTSD, depression and cardiovascular disease in an American Indian tribe was investigated and PTSD was significantly associated with cardiovascular disease, even after controlling for traditional risk factors and major depression. In contrast, the association of major depression with cardiovascular disease was not significant after accounting for both traditional risk factors and PTSD [21]. The independent influence of depression on the association with somatic disease is not possible to investigate in our study, since all migrants suffering from PTSD also suffered from comorbid depression. Furthermore, data in our study was not sufficient to control for relevant risk factors (e.g., smoking, lack of exercise, etc.) for each diagnostic categories. The effect of region of origin and legal basis of obtaining residency on somatic comorbidity among migrants, was also investigated in a study of immigrants in Norway [46] where being immigrant, regardless of area of origin, was negatively associated with multi-morbidity compared with being Norwegian-born. Our study suggests differences in the risk of having somatic comorbidity according to region of origin; thus migrants with PTSD and depression from Asia and North Africa had a significantly higher risk of several somatic diseases compared with migrants without PTSD and depression. An other study of immigrants in Norway [41] also found higher multi-morbidity among refugees, compared with family reunified immigrants. Refugee versus family reunified status was of less importance in our study, potentially due to the small sample size.

We found a significantly higher rate of rheumatic and neurological diseases among patients with PTSD and depression in the present study. Rheumatic and neurological diseases including chronic pain and headache are known comorbidity to individuals suffering from PTSD including migrants suffering from PTSD [13, 14, 47]. The fundamental mechanisms underlying the high comorbidity between PTSD and these symptoms are in general poorly understood [17]. Somatic comorbidity, e.g., rheumatic and various neurological diseases are well-described in individuals who have experienced torture [14, 47, 48]. An unknown number of migrants with PTSD and depression in this present study have been exposed to torture, which may explain the higher rates of rheumatic and neurological diseases including chronic pain, headache and paroxysmal disorders.

### Methodological strengths and limitations

The present study is one of the largest register-based studies exploring somatic comorbidities among migrants diagnosed with PTSD and depression. Furthermore, comorbidity was investigated for a variety of somatic diagnostic categories.

Several factors may have influenced the results of this study. First, the result of this study might have been under influence of selection bias when identifying patients through CTP, as patients have to present to the public healthcare system in order to be referred to CTP. A large proportion of the patients treated at CTP have been referred by general a practitioner. Thus, patients suffering from PTSD and depression in this study have already had contact with the health system and may therefore be more likely to be diagnosed with somatic comorbidity before referred to CTP for treatment. Furthermore, patients at CTP might be more likely to seek their family doctor with both emotional and physical complaints and might therefore to a higher degree be referred to somatic examination. This could mean that disease rates for migrants without PTSD and depression were underestimated due to detection bias. However, we need more studies of health seeking behaviours among migrants with and without PTSD and depression to reach any conclusions. Second, our data is based on public hospital registries and we cannot exclude that a minority of patients with PTSD and depression have been refereed to care in a private rehabilitation clinic, which may have underestimated our results slightly. Third, detection of somatic comorbidity might be increased for patients at CTP as they occasionally undergo a general health examination before being referred to CTP and subsequently have regular contact with a psychiatrist during six months’ treatment, resulting in possible detection bias regarding somatic disorders. However, since the treatment period is rather short compared to the follow up period we do not consider this a major concern. Fourth, all registers, except for NDR, used in this study do not include primary health care. Most Danish patients are diagnosed and treated in primary care. Hence, many chronic diseases such as diabetes mellitus, cardiovascular diseases, chronic obstructive pulmonary disease, osteoporosis, asthma and allergy and rheumatic diseases are mainly treated in primary care [49]. The extent of somatic comorbidity is, therefore, most likely underestimated. Nonetheless, the underestimation is probably equally distributed between the two migrant groups. Further, while angina pectoris, acute myocardial infarction and chronic ischemic disease, atrial fibrillation, viral hepatitis, diabetes mellitus and disorder in the thyroid gland are considered more difficult to misdiagnose due to more or less accurate clinical and biochemical tests, intestinal infectious diseases and other viral diseases are less specific and include a wide range of different conditions. The validity of the diagnoses in the present study may therefore differ by diagnostic category. Fifth, some psychiatric disorders are also mainly treated in primary care and, therefore, not registered in PCR or NPR, and some migrants with an undiagnosed psychiatric disorder or a psychiatric disorder treated in primary care could have been included in the comparison group *no psychiatric disorder,* potentially weakening the association of PTSD, depression and somatic disease. In addition, due to language and cultural barriers, migrants may experience poorer access to healthcare resulting in an underestimation of the psychiatric diagnoses [50]. Sixth, in this study, data from CTP was limited to the psychiatric diagnoses PTSD and depression. Other psychiatric diagnoses, including other anxiety disorders and somatization disorder, were not included. Whether the somatic diagnoses are a real expression of a higher incidence somatic comorbidity or part of a somatization disorder, a somatic component of the PTSD/depression disorder, caused by other well-known risk factors or a combination thereof is unknown and data are not sufficient to pursue this theory. Seventh, data on well-documented risk factors for developing somatic diseases, such as smoking, were not available in this study. It was therefore, not possible to investigate the influence of certain risk factors on somatic disease in the two migrant groups, including risk factors related to environmental exposures and possibly explaining some of the differences related to region of origin. Finally, we assumed that migrants with PTSD and depression had already developed their trauma-related disorder before obtaining residence. However, it is possible that PTSD was developed after arrival in Denmark and/or that somatic disease was developed before the trauma. Nonetheless, two published papers describing the trauma-affected refugees at the time of referral to CTP, from the same period find that the mean number of years since the first trauma was 22 years and the mean number of years with mental health problems as recalled by the patients were 14.7 years, similar to the time of arrival in Denmark. From these studies we expect that the number of years with mental health problems is substantial when admitted to treatment [51, 52].

## Conclusion

Migrants with PTSD and depression had a significantly higher rates of somatic comorbidity compared with migrants without a diagnosed psychiatric disorder. The rates were significantly higher in ten out of the fifteen diagnostic categories after adjusting and was especially high for infectious, neurological and pulmonary diseases. Our results further suggest difference in the rates of somatic comorbidity according to region of origin and according to the legal ground of obtaining residency. Thus, our study indicates that preventive and treatment services should pay special attention to improve the overall health of migrants with PTSD and depression. Further, the understanding, examination and treatment of migrants suffering from PTSD and depression should involve a skilled cross-disciplinary approach, including expertise from somatic clinicians. Earlier identification in the asylum centres of vulnerable migrants suffering from PTSD and depression might also improve the overall health and even prevent the development of somatic disease. More studies are warranted exploring the exact contribution of PTSD to the elevated risk of developing different somatic comorbidities and looking into the mechanisms associated with PTSD and somatic disease.

## Abbreviations

CRS: The Danish civil registration system
CTP: The competence centre for transcultural psychiatry
DM: Diabetes mellitus
DST: Statistics Denmark
IR: Incidence rates
IRR: Incidence rate ratio
NDR: The Danish national diabetes register
NPR: The Danish national patient registry
PCR: The Danish psychiatric central register
PIN: Personal identification number
PTSD: Posttraumatic stress disorder
